# Supplementary material for: Unpacking the Public Health Triad of Social Inequality in Health, Health Literacy, and Quality of Life—A Scoping Review of Research Characteristics
Source: Int J Environ Res Public Health. 2023 Dec 27;21(1):36. doi: 10.3390/ijerph21010036 (PMC10815593; doi:10.3390/ijerph21010036)
Supplement: Supplementary file 1 [file ijerph-21-00036-s001.zip › ijerph-2716598 Table S2.pdf]

Supplementary Table S2. Holmen et al. Unpacking the public health triad of social inequality in health, health literacy and quality of life - a scoping review of research characteristics

Search string from Medline

OVID Medline

1	Health Literacy/
2	(health adj5 (literac* or literate* or illitera*)).mp.
3	((health adj2 competen*) or (medical adj5 (literac* or literate*)) or (understand* adj4 (medical* or health*) adj2 information)).mp.
4	1 or 2 or 3
5	Quality of Life/
6	personal satisfaction/
7	((quality adj2 life) or QOL or HRQL or ((life or personal) adj2 satisf*)).mp. [mp=title, abstract, original title, name of substance word, subject heading word, floating sub-heading word, keyword heading word, organism supplementary concept word, protocol supplementary concept word, rare disease supplementary concept word, unique identifier, synonyms]
8	5 or 6 or 7
9	Social Determinants of Health/
10	health inequities/ or health status disparities/
11	exp Socioeconomic Factors/
12	health services accessibility/ or health equity/ or right to health/ or universal health care/ or healthcare disparities/
13	((health* adj5 (ineq* or disparit* or equit* or status* or service* access*)) or ((social or structural*) adj3 determinant*) or socioeconomic or economic or SES or poverty or (social adj5 (gradient* or class* or mobility or factor*))).mp.
14	9 or 10 or 11 or 12 or 13
15	4 and 8 and 14
