# Supplementary material for: Unpacking the Public Health Triad of Social Inequality in Health, Health Literacy, and Quality of Life—A Scoping Review of Research Characteristics
Source: Int J Environ Res Public Health. 2023 Dec 27;21(1):36. doi: 10.3390/ijerph21010036 (PMC10815593; doi:10.3390/ijerph21010036)
Supplement: Supplementary file 1 [file ijerph-21-00036-s001.zip › ijerph-2716598 Table S3.pdf]

Supplementary Table S3. Characteristics of the included citations Holmen et al.

**References, alphabetically**	**Aim of the study**	**Methods**			**Participants and context**		
Author, year, country	*As reported by the authors*	*Methods*	*Study design*	*Publication type*	*Participants*	*Our categorization of the population*	*Our categorization of context*
(Albus, 2018), Germany	To comment on a multinational study on the association of health literacy health literacy with sociodemographic characteristics in patients with familial hypercholesterolemia.	NA	NA	Orientational report. Editorial. Full paper.	The general population	General population	National/ International
(Alruthia et al., 2020), Saudi Arabia	To examine the association between the physical, mental, or psychological, social, and environmental domains of health-related quality of life with the patients' trust in their primary care physicians while controlling for several sociodemographic and clinical factors.	Quantitative	Cross-sectional	Full paper	Patients with diabetes	Patients	Regional/ District
(Amoah & Phillips, 2018), Ghana	To investigate how health literacy may operate in the context of universal health coverage (UHC), especially the role of health literacy in the relationships between elements of UHC such as access to health care and health insurance.	Quantitative	Cross-sectional	Full paper	The general population in rural and urban districts	Other	Regional/ District
(An, Moon, & Cha, 2019), South Korea	To identify the risk factors and provide data for public health policy in support of Chinese immigrants, this study examined health literacy, depression, and immigrant stress in Chinese immigrants living in South Korea.	Quantitative	Cross-sectional	Full paper	Chinese immigrants in South Korea	Immigrants/minorities	Community
(Angner, Miller, Ray, Saag, & Allison, 2010), USA	To explore the relationship between health literacy and happiness while controlling for sociodemographic factors and health status in a community-based sample of older adults.	Quantitative	Cross-sectional	Full paper	Community-dwelling older primary care patients	Patients	Community
(Apter et al., 2015), USA	To describe how to compare portal training and home visits by a community health worker with an active control of portal training (without home visits). Baseline data of the first participants.	Quantitative	Cross-sectional findings and a protocol for an RCT	Protocol, full paper	Adult African/American and Hispanic/Latino patients with uncontrolled asthma from low-income urban neighborhoods	Patients	Community
(Apter et al., 2017), USA	To describe the planning, design, methodology, challenges, and baseline results of this ongoing randomized controlled trial Helping Asthma Patients 2 (HAP2). Mediators and moderators of the Patient	Quantitative	Cross-sectional findings and a protocol for an RCT	Protocol, full paper	Low-income asthma patients	Patients/Inpovered populations	Regional/ District

References, alphabetically	Aim of the study	Methods			Participants and context		
Author, year, country	*As reported by the authors*	*Methods*	*Study design*	*Publication type*	*Participants*	*Our categorization of the population*	*Our categorization of context*
	Advocate (PA) asthma outcome relationship are examined along with cost-effectiveness.						
(Asare et al., 2019), USA	To assess cancer patients' and survivors' physical activity behaviors by race/ethnicity.	Quantitative	Cross-sectional	Full paper	Black cancer survivors	Patients/minorities	National/ International
(Batista, Lawrence, & Sousa, 2017), Brazil	To investigate the association between critical and communicative oral health literacy and oral health outcomes (status, oral health-related quality of life and practices) in adults.	Quantitative	Cross-sectional	Full paper	The adult population in Piracicaba, Brazil	General population	Regional/ District
(Blancafort Alias et al., 2021), Spain	To assess the effectiveness of a group-based intervention to improve self-perceived health as indicator of health inequality.	Quantitative	RCT	Full paper	Older adults with low self-perceived health	Other	Community
(Bragard, Coucke, Petre, Etienne, & Guillaume, 2017), Belgium	To present health literacy's different dimensions, the difficulties associated with its evaluations, and the development of effective interventions, as well as the many challenges that remain to be met in this field [French].	NA	NA	Orientational report. Statement. Full paper	The general Belgian population	General population	National/ International
(Clarke & Voss, 2016), Republic of South Africa	To determine whether a community-based, multidisciplinary team consisting of home-based caregivers and supervised students could improve the functional status and quality of life of patients living with chronic obstructive pulmonary disease (COPD) in a low-income, peri-urban setting in South Africa.	Quantitative	Pre-posttest	Full paper	Low-income COPD patients in South Africa	Patients/Inpovered populations	Community
(Curtis, Wolf, Weiss, & Grammer, 2012), USA	To examine the independent contributions of socioeconomic status and health literacy in explaining asthma disparities.	Quantitative	Cohort	Full paper	Adult patients (18-40 years) with persistent asthma in Chicago	Patients	Regional/ District
(de Vries, Buitrago, Quitian, Wiesner, & Castillo, 2018), Colombia	To present the most currently available data on real access to high quality diagnostic and curative and palliative care in Colombia, a middle-income country with relatively recent large healthcare reforms.	NA	NA	Orientational report. Full paper.	Cancer patients in general	Patients	Regional/ District
(Durand et al., 2021), USA	To understand how to support women across socioeconomic strata in making breast cancer surgery choices.	Quantitative	RCT	Full paper	Women undergoing breast cancer surgery	Patients	Regional/ District
(Ernsting et al., 2017), Germany	To explore the extent of smartphone and health app use, sociodemographic, medical, and behavioral correlates of smartphone and health app use, and	Quantitative	Cross-sectional study	Full paper	The general adult German population	General population	National/ International

**References, alphabetically**	**Aim of the study**	**Methods**			**Participants and context**		
Author, year, country	*As reported by the authors*	*Methods*	*Study design*	*Publication type*	*Participants*	*Our categorization of the population*	*Our categorization of context*
	associations of the use of apps and app characteristics with actual health behaviors.						
(Faruqi, Stocks, Spooner, el Haddad, & Harris, 2015), Australia	To evaluate the implementation and effectiveness of using practice nurses in the role of 'prevention navigators' to support obese patients with low health literacy to better manage their weight.	Quantitative	Protocol for intervention	Protocol, full paper	Patients with obesity	Patients	Community
(Fung et al., 2016), China	To investigate, analyze and evaluate the effect of the Health Empowerment Program on the health, health-related quality of life, lifestyle, and health-seeking behaviors among working poor families.	Quantitative	Protocol for prospective cohort	Protocol, full paper	Poor working families with small children in China	Inpovered populations	Community
(Ghisi, Chaves, Britto, & Oh, 2018), Canada	To identify health literacy screening instruments available to coronary artery disease patients; describe the prevalence of low health literacy; explore the predictors of low health literacy; and identify the association between health literacy, health behaviors, and outcomes among these patients.	Review	Review	Full paper	Patients with coronary artery disease	Patients	Community
(Gibbs, Guarnieri, Chu, Murdoch, & Asif, 2021), USA	Conceptualize a systematic approach of the development of a tailored integrated community and care team to develop a partnership in assisting senior adults with multiple chronic conditions	Review	Review	Full paper	Vulnerable older adults with multiple chronic conditions	Patients	Community
(Goss et al., 2021), Ireland	To explore the perceptions of socially disadvantaged Irish adolescents in relation to health literacy and related behaviors and utilize these data to develop relevant vignettes.	Mixed methods	Convergent mixed methods	Full paper	Disadvantaged adolescents	Inpovered populations	Community
(Graham et al., 2021), USA	To examine the effect of patient-reported quality of life and psychosocial determinants of health on unplanned hospital readmissions in a surgical population.	Quantitative	Prospective cohort	Full paper	Veterans undergoing elective inpatient surgery	Patients	Regional/ District
(Griech & Skrzat, 2020), USA	To discuss health literacy and its intersectional relationship with the Social Determinants of Health in patients with cardiovascular disease and to present recommendations for physical therapists to reduce health inequities related to limited health literacy.	NA	NA	Orientational report. Clinical perspective. Full paper	Patients with cardiovascular disease	Patients	Regional / District
(Guhl et al., 2019), USA	To examine the association between income and health-related quality of life in a cohort with prevalent atrial fibrillation.	Quantitative	Cohort	Full paper	Patient with atrial fibrillation	Patients	Regional/ District

**References, alphabetically**	**Aim of the study**	**Methods**			**Participants and context**		
Author, year, country	*As reported by the authors*	*Methods*	*Study design*	*Publication type*	*Participants*	*Our categorization of the population*	*Our categorization of context*
(Hardgraves, Henry, & Patton, 2021), USA	To describe attitudes, expectations, knowledge, and intentions related to oral health issues from the experiences of older adults living independently in a largely rural south-central state.	Qualitative	Interview	Full paper	Older adults	Other	Community
(Harsch et al., 2021), Afghanistan	To analyze the relationship between health literacy, quality of life and spiritual and religious beliefs.	Quantitative	Cross-sectional	Full paper	The general population of Gahnzi in Afghanistan	General population	Community
(Hickey et al., 2018), USA	To evaluate the racial and ethnic differences in health literacy and coexisting self-reported quality of life in an older adult, ethnically diverse, disadvantaged population with chronic comorbidities.	Quantitative	Cross-sectional study	Conference abstract, protocol	Older adults, ethnically diverse, disadvantaged population with chronic comorbidities	Patients	Community
(Irwin, 2018), USA	To address the vulnerabilities experienced by patients with serious mental illness and cancer from disadvantaged communities by developing a targeted collaborative care intervention.	Quantitative	Intervention development	Conference abstract	Individuals with serious mental illness and cancer	Patients	Regional / District
(Jamieson, Divaris, Parker, & Lee, 2013), Australia/ USA	To compare oral health literacy levels between two profoundly disadvantaged groups, Indigenous Australians, and American Indians, and to explore differences in sociodemographic, dental service utilization, self-reported oral health indicators, and oral health-related quality of life correlates of oral health literacy among the above.	Quantitative	Cross-sectional	Full paper	Indigenous Australian and American Indians	Immigrants/minorities	Regional/ District
(Johnson et al., 2015), Australia	To report on selected baseline clinical and socioeconomic data from this cluster randomized study aiming to deliver a program of integrated community-based intensive chronic disease management for Indigenous people in remote communities in Australia.	Quantitative	Cross-sectional	Full paper	Indigenous Australian adults with diabetes	Patients/Immigrants/minorities	Community
(Katzmarzyk et al., 2018), USA	To test the effectiveness of a pragmatic, high-intensity lifestyle-based obesity treatment program delivered within primary care among an underserved population.	Quantitative	RCT	Full paper	Low income African Americans who are overweight	Inpovered populations and immigrants/minorities	Community
(Kim et al., 1999), USA	To present baseline of Veterans Administrations (VA) Cancer of the Prostate Outcomes Study (CaPOS) of lower socioeconomic status patients.	Quantitative	Cross-sectional	Full paper	Prostate cancer patients with low socioeconomic status	Patients/ Inpovered populations	National/ International

References, alphabetically	Aim of the study	Methods			Participants and context		
Author, year, country	*As reported by the authors*	*Methods*	*Study design*	*Publication type*	*Participants*	*Our categorization of the population*	*Our categorization of context*
(Kim et al., 2001), USA	To evaluate (i) knowledge, level of satisfaction, and treatment preferences and intentions of men newly diagnosed with prostate cancer after participation in a CD-ROM shared decision-making program; and (ii) the relationship between prostate cancer knowledge and health literacy.	Quantitative	Pre-posttest of intervention	Full paper	Patients with prostate cancer	Patients	Regional/ District
(Kuehnert et al., 2022), USA	To establish conceptual consensus for what social determinants of health mean for nursing, with emphasis on health policies that advance planetary health equity and improve planetary health-related quality of life.	NA	NA	Orientational report. Consensus paper. Full paper	The general population	General population	National/ International
(Ladak et al., 2020), Pakistan	To explore parental perspectives on the influence of socio-cultural factors and environmental resources on the health-related quality of life of children and adolescents after congenital heart disease surgery.	Qualitative	Interviews	Full paper	Children and adolescents who had congenital heart surgery in a low-income country	Patients/ Inpovered populations	Regional/ District
(Lang, 2021), USA	To describe self-care management for vulnerable populations.	Quantitative	Cross-sectional	Dissertation	Homeless people with diabetes and hypertension	Patients/ Inpovered populations	Community
(Langton, 2012), USA	To examine two research questions with implications for child health and children's health insurance coverage and a third that may help develop more reliable measurement techniques to aid in future research and practice.	Multimethod	Multimethod cross-sectional and longitudinal dataset	Dissertation	Children with cancer and their parents	Patients	Community
(Lowe & Nobriga, 2021), USA	To explore the beliefs, access, and motivations of individuals with head and neck cancer living in a rural community in the United States, regarding their speech and swallowing deficits.	Qualitative	Interviews	Full paper	Individuals with head and neck cancer and their caregivers	Patients and caregivers	Regional/ District
(Macabasco-O'Connell et al., 2011), USA	To examine the relationship between literacy and heart failure-related quality of life, and to explore whether literacy-related differences in knowledge, self-efficacy, and/or self-care behavior explained the relationship.	Quantitative	Cross-sectional	Full paper	Patients with heart failure	Patients	Regional/ District
(Maliski, Connor, Oduro, & Litwin, 2011), USA	To explore links between access to care and quality of life for underserved men with prostate cancer through a literature review.	Review	Review	Full paper	Underserved men with prostate cancer	Patients/ Inpovered populations	National/ International

References, alphabetically	Aim of the study	Methods			Participants and context		
Author, year, country	*As reported by the authors*	*Methods*	*Study design*	*Publication type*	*Participants*	*Our categorization of the population*	*Our categorization of context*
(McDougall et al., 2019), New Mexico	To investigate the relationship between socioeconomic characteristics and health-related quality of life in a diverse group of colorectal cancer survivors.	Quantitative	Cross-sectional	Full paper	Colorectal cancer survivors	Patients	Regional/ District
(Merriman, Ades, & Seffrin, 2002), USA	To discuss health literacy in the information age.	NA	NA	Orientational report. Editorial. Full paper	Cancer patients and families in the US with low health literacy skills	Patients	National/ International
(Meyers et al., 2014), USA	To examine how health literacy and other social determinants affect the quality-of-care transitions from hospital to home, as well as subsequent health outcomes including medication use, functional status, health-related quality of life, unplanned health care utilization, and mortality.	Quantitative	Protocol for prospective cohort	Full paper	Hospitalized patients with acute coronary disease	Patients	Community
(Miller, Cage, Nowacki, Jackson, & Modlin, 2018), USA	To highlight findings of a study conducted with minority men focusing on the association between health literacy and health-related quality of life.	Quantitative	Cross-sectional	Full paper	Minority men	Immigrants/minorities	Community
(Myaskovsky et al., 2011), USA	To examine the association of race and cultural factors with quality of life factors (participation, life satisfaction, perceived health status) in people with spinal cord injury.	Quantitative	Cross-sectional	Full paper	Persons With Spinal Cord Injury	Patients	Regional/ District
(Omachi, Sarkar, Yelin, Blanc, & Katz, 2011), USA	To examine the associations between health literacy and both outcomes and health status in COPD.	Quantitative	Cross-sectional	Conference abstract	Patients with COPD	Patients	National/ International
(Ownby et al., 2017), USA	To develop a computer-delivered mobile intervention that will provide individuals with chronic conditions the necessary information to cope with their conditions.	Mixed methods	Interview-based development of an intervention and protocol for a randomized controlled trial	Protocol, full paper	Individuals with chronic conditions	Patients	Community
(Park et al., 2018), Korea	To investigate the effects of medication adherence and health literacy on health-related quality of life and identify factors associated with health-related quality of life in vulnerable older people with hypertension.	Quantitative	Cross-sectional	Full paper	Low-income older people with hypertension in South Korea	Patients/ Inpovered populations	Community

**References, alphabetically**	**Aim of the study**	**Methods**			**Participants and context**		
Author, year, country	*As reported by the authors*	*Methods*	*Study design*	*Publication type*	*Participants*	*Our categorization of the population*	*Our categorization of context*
(Prihanto et al., 2021), Indonesia	To assess the effect of health literacy on the QoL among Indonesian undergraduate university students when socioeconomic considerations are taken into account	Quantitative	Cross-sectional	Full paper	University students in Indonesia	Other	Community
(Rak, 2012), USA	To investigate the prevalence of low health literacy in individuals with diabetes, and to examine associations between health literacy and certain domains of quality of life.	Quantitative	Cross-sectional	Dissertation	Persons with diabetes in Michigan	Patients	Regional/ District
(Reid et al., 2021), USA	To determine the prevalence of limited health literacy and to explore the relationship between adolescents' health literacy status and energy balance related behaviors, including nutrition, physical activity, sedentary activity, and sleep, with the secondary objectives being to explore how health literacy is related to BMI and school related quality of life among adolescents.	Quantitative	Cross-sectional	Full paper	Rural Appalachian middle school students in Virginia	Other	Community
(Rijken & van der Heide, 2019), Netherlands	To examine whether subgroups of people with multimorbidity could be distinguished based on their needs, and profiled these subgroups according to medical complexity and the availability of personal resources.	Quantitative	Cross-sectional	Full paper	People with multimorbidity	Patients	Community
(Roberto et al., 2018), Brazil	To investigate factors associated with the lack of access to information on oral health among adults [Portuguese].	Quantitative	Cross-sectional	Full paper	Individuals from rural and urban areas	Other	Community
(Rozier, 2012), USA	To review some oral health initiatives and their outcomes with a focus on youth.	NA	NA	Orientational report.<br>Issue brief.<br>Full paper.	Young dental patients in North Carolina	Patients	National/ International
(Schaffler et al., 2018), Canada/ review	To review self-management interventions in populations with low income or low health literacy and synthesize the efficacy of these interventions.	Review	Systematic review	Full paper	Low-income or low-health literacy people with chronic illness	Patients/ Inpovered populations	National/ International
(Scheuer, 2018), Denmark	To examine whether the Danish Cancer Society's nation-wide Patient Navigation Project improves quality of life and health literacy among socially vulnerable cancer patients.	Quantitative	Pre-test post-test	Conference abstract	Socially vulnerable cancer patients	Patients/ Inpovered populations	Community

**References, alphabetically**	**Aim of the study**	**Methods**			**Participants and context**		
Author, year, country	*As reported by the authors*	*Methods*	*Study design*	*Publication type*	*Participants*	*Our categorization of the population*	*Our categorization of context*
(Simon, Li, & Dong, 2014), USA	To examine health literacy level among community-dwelling Chinese older adults in the greater Chicago area, and examine the sociodemographic, family position, and health-related correlates of health literacy among this population.	Quantitative	Cohort	Full paper	Older adult Chinese in the greater Chicago area	Immigrants/minorities	Community
(Stormacq, Wosinski, Boillat, & Den Broucke, 2020), Europe and USA, review	To identify and synthesize the best available evidence on the effectiveness on health-related outcomes of health literacy interventions for enabling socioeconomically disadvantaged people living in the community to access, understand, appraise and apply health information; and ii) to identify components of health literacy interventions associated with improved health-related outcomes.	Review	Systematic review	Review, systematic, full paper	Socially or socioeconomically disadvantaged adults	Inpovered populations	Community
(Talmage, Figueroa, & Wolfersteig, 2018), USA	To describe the connections between social determinants and quality of life as elucidated from a large-scale community health needs assessment conducted in Maricopa County, Arizona.	Qualitative	Focus groups	Full paper	The general population of Maricopa County in Arizona	General population	Community
(Tan et al., 2019), Italia, Netherlands, Spain, France, UK	To evaluate the benefits of offering the chronic disease self-management program intervention to the target population.	Quantitative	Protocol for intervention	Protocol, full paper	Adults with a chronic condition and their caregivers in a low socioeconomic position	Patients	Community
(Todorovic et al., 2019), Bosnia	To assess health literacy and its association with sociodemographic variables, self-perception of health and the presence of chronic conditions in primary healthcare setting.	Quantitative	Cross-sectional	Full paper	Patients with a family physician in primary health care settings	Patients	Community
(Uddin, Bhar, Al Mahmud, & Islam, 2017), Bangladesh	To report the awareness, knowledge, attitudes, and practice of mental disorders; estimate the prevalence of and risk factors for psychological distress; measure association of psychological distress and other sociodemographic factors with quality of life and test the feasibility to use Kessler 10-item (K10) and WHO Quality of Life-BREF (WHOQOL-BREF) questionnaires in rural Bangladesh for measuring psychological distress and quality of life.	Quantitative	Protocol for a prospective cohort	Full paper	Adults and older adults in the rural Bangladesh population	Inpovered populations	Community

References, alphabetically	Aim of the study	Methods			Participants and context		
Author, year, country	*As reported by the authors*	*Methods*	*Study design*	*Publication type*	*Participants*	*Our categorization of the population*	*Our categorization of context*
(Virlée, van Riel, & Hammedi, 2020), Belgia	To develop a better understanding of how online health community members with different health literacy levels benefit from their participation, through the analysis and comparison of their resource integration processes.	Multimethod	Multimethod (cross-sectional and interview)	Full paper	Online health community participants	Other	Community
(Walker, Gebregziabher, Martin-Harris, & Egede, 2014), USA	To investigate the independent effects of socioeconomic and psychological social determinants of health on diabetes knowledge, self-care, diabetes outcomes and quality of life.	Quantitative	Cross-sectional	Full paper	Adults with type 2 diabetes	Patients	Community
(Wang et al., 2013), China	To examine the relationship between health literacy and health-related quality of life as well as relationship differentials by ethnicity among rural women from a Chinese poor minority area.	Quantitative	Cross-sectional	Full paper	Vulnerable minority women aged 23-57 in rural China	Immigrants/minorities	Community
(Wang, Kane, Xu, & Meng, 2015), China	To examine how health literacy might modify the association between chronic disease and their health-related quality of life impacts.	Quantitative	Cross-sectional	Full paper	Vulnerable minority women aged 23-57 in rural China	Inpovered populations	Community
(Washington, Curtis, Waite, Wolf, & Paasche-Orlow, 2018), USA	To characterize the longitudinal effect of race/ethnicity on childhood asthma outcomes, and to what extent caregiver health literacy, education level, and asthma knowledge mediate these associations.	Quantitative	Cross-sectional cohort	Full paper	Children with asthma and their families in Chicago	Patients	Community
(Webb, 2020), USA	To understand the impact of social determinants of health on a chronically transfused population with sickle cell disease, as well as available screening tools. Recognize the role of health literacy in complex medical decision-making and universal communication strategies to improve comprehension in patients of all health literacy levels. Identify health-related quality of life measures that are affected by chronic transfusion therapy.	Case study	Case study	Case study, full paper	Patients receiving chronic transfusion therapy, case study of a 2-year-old with sickle-cell and her mother	Patients	Regional/ District
(White, Hall, & Johnson, 2014), USA	To understand community beliefs and knowledge of environmental health risks, determine community levels of trust in federal/local agencies and community groups, and identify strategies for mobilizing residents using environmental health messages and environmental health education programs.	Qualitative	Focus groups	Full paper	People in communities with associated poverty	Inpovered populations	Community

References, alphabetically	Aim of the study	Methods			Participants and context		
Author, year, country	*As reported by the authors*	*Methods*	*Study design*	*Publication type*	*Participants*	*Our categorization of the population*	*Our categorization of context*
(Wong Min et al., 2022), Malaysia	To investigate factors associated with depression and anxiety symptoms and quality of life among Malaysia's multiethnic urban lower-income communities.	Quantitative	Cross-sectional survey	Full paper	Low-income dwellers in Malaysia	Inpovered populations	Community
(Xiao, Lee, & Liu, 2020), USA	To examine the interrelationships among demographics, health literacy, self-perceived health status, and quality of life using structural equation modeling.	Quantitative	Cross-sectional	Full paper	Korean and Vietnamese immigrants in the US	Immigrants/minorities	Community
(Xu, Zhou, Wong, & Wang, 2021), China	To assess the relationship between patients' eHealth literacy and their socioeconomic determinants and to investigate the association between patients' eHealth literacy and their satisfaction with shared decision-making and well-being.	Quantitative	Cross-sectional	Full paper	Patients in general in China	Patients	Regional / District
(Aaby, Friis, Christensen, & Maindal, 2020), Denmark	To examine the associations between health literacy and participation in cardiac rehabilitation and health literacy and health-related quality of life.	Quantitative	Cross-sectional	Full paper	adult cardiac rehabilitation patients	Patients	Community

Full citations of the included reports

Albus, C. (2018). Health literacy: Is it important for cardiovascular disease prevention? *European Journal of Preventive Cardiology, 25(9)*, 934-935. doi:http://dx.doi.org/10.1177/2047487318770519

Alruthia, Y., Sales, I., Almalag, H., Alwhaibi, M., Almosabhi, L., Albassam, A. A., . . . Asiri, Y. (2020). The relationship between health-related quality of life and trust in primary care physicians among patients with diabetes. *Clinical Epidemiology, 12*, 143-151. doi:http://dx.doi.org/10.2147/CLEP.S236952

Amoah, P. A., & Phillips, D. R. (2018). Health literacy and health: rethinking the strategies for universal health coverage in Ghana. *Public health, 159*, 40-49. doi:https://dx.doi.org/10.1016/j.puhe.2018.03.002

An, J. Y., Moon, H., & Cha, S. (2019). Health literacy, depression, and stress among Chinese immigrants in South Korea. *Public Health Nursing, 36*(5), 603-614. doi:https://dx.doi.org/10.1111/phn.12632

Angner, E., Miller, M. J., Ray, M. N., Saag, K. G., & Allison, J. J. (2010). Health literacy and happiness: A community-based study. *Social Indicators Research, 95*(2), 325-338. doi:https://dx.doi.org/10.1007/s11205-009-9462-5

Apter, A. J., Bryant, S., Morales, K. H., Wan, F., Hardy, S., Reed-Wells, S., . . . Localio, R. (2015). Using IT to improve access, communication, and asthma in African American and Hispanic/Latino Adults: Rationale, design, and methods of a randomized controlled trial. *Contemporary Clinical Trials, 44*, 119-128. doi:http://dx.doi.org/10.1016/j.cct.2015.08.001

Apter, A. J., Morales, K. H., Han, X., Perez, L., Huang, J., Ndicu, G., . . . Schwartz, J. S. (2017). A patient advocate to facilitate access and improve communication, care, and outcomes in adults with moderate or severe asthma: Rationale, design, and methods of a randomized controlled trial. *Contemporary Clinical Trials, 56*, 34-45. doi:https://dx.doi.org/10.1016/j.cct.2017.03.004

Asare, M., McIntosh, S., Culakova, E., Alio, A., Umstattd Meyer, M. R., Kleckner, A. S., . . . Kamen, C. S. (2019). Assessing Physical Activity Behavior of Cancer Survivors by Race and Social Determinants of Health. *International Quarterly of Community Health Education, 40*(1), 7-16. doi:10.1177/0272684X19857427

Batista, M. J., Lawrence, H. P., & Sousa, M. (2017). Oral health literacy and oral health outcomes in an adult population in Brazil. *BMC Public Health, 18*(1), 60. doi:https://dx.doi.org/10.1186/s12889-017-4443-0

Blancafort Alias, S., Monteserin Nadal, R., Moral, I., Roque Figols, M., Rojano, I. L. X., & Coll-Planas, L. (2021). Promoting social capital, self-management and health literacy in older adults through a group-based intervention delivered in low-income urban areas: results of the randomized trial AEQUALIS. *BMC Public Health, 21*(1), 84. doi:https://dx.doi.org/10.1186/s12889-020-10094-9

Bragard, I., Coucke, P. A., Petre, B., Etienne, A. M., & Guillaume, M. (2017). [Health literacy, a way to reduce social health inequalities]. *Revue Medicale de Liege, 72*(1), 32-36. Retrieved from https://ovidsp.ovid.com/ovidweb.cgi?T=JS&CSC=Y&NEWS=N&PAGE=fulltext&D=med14&AN=28387075

Clarke, H., & Voss, M. (2016). The role of a multidisciplinary student team in the community management of chronic obstructive pulmonary disease. *Primary Health Care Research & Development, 17*(4), 415-420. doi:https://dx.doi.org/10.1017/S1463423616000013

Curtis, L. M., Wolf, M. S., Weiss, K. B., & Grammer, L. C. (2012). The impact of health literacy and socioeconomic status on asthma disparities. *Journal of Asthma, 49*(2), 178-183. doi:https://dx.doi.org/10.3109/02770903.2011.648297

de Vries, E., Buitrago, G., Quitian, H., Wiesner, C., & Castillo, J. S. (2018). Access to cancer care in Colombia, a middle-income country with universal health coverage. *Journal of Cancer Policy, Part B. 15*, 104-112. doi:http://dx.doi.org/10.1016/j.jcpo.2018.01.003

Durand, M. A., Yen, R. W., O'Malley, A. J., Schubbe, D., Politi, M. C., Saunders, C. H., . . . Elwyn, G. (2021). What matters most: Randomized controlled trial of breast cancer surgery conversation aids across socioeconomic strata. *Cancer, 127*(3), 422-436. doi:10.1002/cncr.33248

Ernsting, C., Dombrowski, S. U., Oedekoven, M., J.L, O. S., Kanzler, M., Kuhlmey, A., & Gellert, P. (2017). Using Smartphones and Health Apps to Change and Manage Health Behaviors: A Population-Based Survey. *Journal of medical Internet research, 19(4)*, e101. doi:http://dx.doi.org/10.2196/jmir.6838

Faruqi, N., Stocks, N., Spooner, C., el Haddad, N., & Harris, M. F. (2015). Research protocol: Management of obesity in patients with low health literacy in primary health care. *BMC Obesity, 2(1) (no pagination)*. doi:http://dx.doi.org/10.1186/s40608-015-0036-6

Fung, C. S. C., Yu, E. Y. T., Guo, V. Y., Wong, C. K. H., Kung, K., Ho, S. Y., . . . Lam, C. L. K. (2016). Development of a Health Empowerment Programme to improve the health of working poor families: Protocol for a prospective cohort study in Hong Kong. *BMJ Open, 6(2) (no pagination)*. doi:http://dx.doi.org/10.1136/bmjopen-2015-010015

Ghisi, G. L. D. M., Chaves, G. S. D. S., Britto, R. R., & Oh, P. (2018). Health literacy and coronary artery disease: A systematic review. *Patient education and counseling, 101(2)*, 177-184. doi:http://dx.doi.org/10.1016/j.pec.2017.09.002

Gibbs, J. F., Guarnieri, E., Chu, Q. D., Murdoch, K., & Asif, A. (2021). Value-based chronic care model approach for vulnerable older patients with multiple chronic conditions. *Journal of Gastrointestinal Oncology, 12*(Suppl 2), S324-S338. doi:https://dx.doi.org/10.21037/jgo-20-317

Goss, H. R., McDermott, C., Hickey, L., Issartel, J., Meegan, S., Morrissey, J., . . . Belton, S. (2021). Understanding disadvantaged adolescents' perception of health literacy through a systematic development of peer vignettes. *BMC Public Health, 21*(1), 1-11. doi:10.1186/s12889-021-10634-x

Graham, L. A., Hawn, M. T., Dasinger, E. A., Baker, S. J., Oriel, B. S., Wahl, T. S., . . . Morris, M. S. (2021). Psychosocial Determinants of Readmission after Surgery. *Medical care, 59(10)*, 864-871. doi:http://dx.doi.org/10.1097/MLR.0000000000001600

Griech, S. F., & Skrzat, J. M. (2020). Roles for Physical Therapists to Address Health Literacy as a Social Determinant of Cardiovascular Disease: A Clinical Perspective. *Cardiopulmonary Physical Therapy Journal, 31(1)*, 29-34. doi:http://dx.doi.org/10.1097/CPT.0000000000000132

Guhl, E., Althouse, A., Sharbaugh, M., Pusateri, A. M., Paasche-Orlow, M., & Magnani, J. W. (2019). Association of income and health-related quality of life in atrial fibrillation. *Open Heart, 6(1) (no pagination)*. doi:http://dx.doi.org/10.1136/openhrt-2018-000974

Hardgraves, V. M., Henry, L. J., & Patton, S. K. (2021). "I'm Gonna Hang on to These As Long As I Can": Examining the Perspectives and Knowledge of Oral Health Issues of Older Adults Living Independently. *American Journal of Lifestyle Medicine*. doi:10.1177/15598276211026842

Harsch, S., Jawid, A., Jawid, M. E., Saboga Nunes, L., Sahrai, D., & Bittlingmayer, U. H. (2021). The relationship of health literacy, wellbeing and religious beliefs in neglected and unequal contexts-Results of a survey study in central Afghanistan. *Health Promotion Journal of Australia, 32 Suppl 1*, 80-87. doi:https://dx.doi.org/10.1002/hpja.419

Hickey, K. T., Masterson Creber, R. M., Reading, M., Sciacca, R. R., Riga, T. C., Frulla, A. P., & Casida, J. M. (2018). Low health literacy: Implications for managing cardiac patients in practice. *Nurse Practitioner, 43*(8), 49-55. doi:https://dx.doi.org/10.1097/01.NPR.0000541468.54290.49

Irwin, K. (2018). Improving cancer care for underserved communities in northeastern Massachusetts: Serious mental illness and cancer. *Psycho-Oncology, 27(Supplement 1)*, 37-38. doi:http://dx.doi.org/10.1002/pon.4622

Jamieson, L. M., Divaris, K., Parker, E. J., & Lee, J. Y. (2013). Oral health literacy comparisons between Indigenous Australians and American Indians. *Community Dental Health, 30*(1), 52-57. Retrieved from https://ovidsp.ovid.com/ovidweb.cgi?T=JS&CSC=Y&NEWS=N&PAGE=fulltext&D=med10&AN=23550508

Johnson, D. R., McDermott, R. A., Clifton, P. M., D'Onise, K., Taylor, S. M., Preece, C. L., & Schmidt, B. A. (2015). Characteristics of Indigenous adults with poorly controlled diabetes in north Queensland: implications for services. *BMC Public Health, 15*, 325. doi:https://dx.doi.org/10.1186/s12889-015-1660-2

Katzmarzyk, P. T., Martin, C. K., Newton, R. L., Jr., Apolzan, J. W., Arnold, C. L., Davis, T. C., . . . Springgate, B. (2018). Promoting Successful Weight Loss in Primary Care in Louisiana (PROPEL): Rationale, design and baseline characteristics. *Contemporary Clinical Trials, 67*, 1-10. doi:https://dx.doi.org/10.1016/j.cct.2018.02.002

Kim, S. P., Bennett, C. L., Chan, C., Chmiel, J., Falcone, D., Knight, S. J., . . . Smith, J. S. (1999). QOL and outcomes research in prostate cancer patients with low socioeconomic status. *Oncology (Williston Park), 13*(6), 823-832; discussion 835. Retrieved from https://ovidsp.ovid.com/ovidweb.cgi?T=JS&CSC=Y&NEWS=N&PAGE=fulltext&D=med4&AN=10378220

Kim, S. P., Knight, S. J., Tomori, C., Colella, K. M., Schoor, R. A., Shih, L., . . . Bennett, C. L. (2001). Health literacy and shared decision making for prostate cancer patients with low socioeconomic status. *Cancer Investigation, 19*(7), 684-691. Retrieved from https://ovidsp.ovid.com/ovidweb.cgi?T=JS&CSC=Y&NEWS=N&PAGE=fulltext&D=med4&AN=11577809

Kuehnert, P., Fawcett, J., DePriest, K., Chinn, P., Cousin, L., Ervin, N., . . . Waite, R. (2022). Defining the social determinants of health for nursing action to achieve health equity: A consensus paper from the American Academy of Nursing. *Nursing Outlook, 70*(1), 10-27. doi:10.1016/j.outlook.2021.08.003

Ladak, L. A., Gallagher, R., Hasan, B. S., Awais, K., Abdullah, A., & Gullick, J. (2020). Exploring the influence of socio-cultural factors and environmental resources on the health related quality of life of children and adolescents after congenital heart disease surgery: parental perspectives from a low middle income country. *Journal of Patientreported Outcomes, 4*(1), 72. doi:https://dx.doi.org/10.1186/s41687-020-00239-0

Lang, L. P. (2021). Self-care management in homeless adults with hypertension. *Dissertation Abstracts International: Section B: The Sciences and Engineering, 82*(4-B), No-Specified. Retrieved from http://ovidsp.ovid.com/ovidweb.cgi?T=JS&PAGE=reference&D=psyc18&NEWS=N&AN=2020-86254-166

Langton, C. E. (2012). Pathways to increasing child health: Implications for policy, research, and practice. *Dissertation Abstracts International Section A: Humanities and Social Sciences, 73*(3-A), 1197. Retrieved from http://ovidsp.ovid.com/ovidweb.cgi?T=JS&PAGE=reference&D=psyc9&NEWS=N&AN=2012-99170-597

Lowe, S. M., & Nobriga, C. V. (2021). Head and Neck Cancer in a Rural U.S. Population: Quality of Life, Coping, Health Care Literacy, and Access to Services. *American journal of speech-language pathology, 30(3)*, 1116-1133. doi:http://dx.doi.org/10.1044/2021_AJSLP-20-00223

Macabasco-O'Connell, A., DeWalt, D. A., Broucksou, K. A., Hawk, V., Baker, D. W., Schillinger, D., . . . Pignone, M. (2011). Relationship between literacy, knowledge, self-care behaviors, and heart failure-related quality of life among patients with heart failure. *Journal of general internal medicine, 26*(9), 979-986. doi:https://dx.doi.org/10.1007/s11606-011-1668-y

Maliski, S. L., Connor, S. E., Oduro, C., & Litwin, M. S. (2011). Access to health care and quality of life for underserved men with prostate cancer. *Seminars in Oncology Nursing, 27*(4), 267-277. doi:https://dx.doi.org/10.1016/j.soncn.2011.07.005

McDougall, J. A., Blair, C. K., Wiggins, C. L., Goodwin, M. B., Chiu, V. K., Rajput, A., & Kinney, A. Y. (2019). Socioeconomic disparities in health-related quality of life among colorectal cancer survivors. *Journal of Cancer Survivorship, 13*(3), 459-467. doi:https://dx.doi.org/10.1007/s11764-019-00767-9

Merriman, B., Ades, T., & Seffrin, J. R. (2002). Health literacy in the information age: Communicating cancer information to patients and families. *Electroanalysis, 52*(3), 130-133. doi:10.3322/canjclin.52.3.130

Meyers, A. G., Salanitro, A., Wallston, K. A., Cawthon, C., Vasilevskis, E. E., Goggins, K. M., . . . Kripalani, S. (2014). Determinants of health after hospital discharge: rationale and design of the Vanderbilt Inpatient Cohort Study (VICS). *BMC health services research, 14*, 10. doi:https://dx.doi.org/10.1186/1472-6963-14-10

Miller, D. B., Cage, J. L., Nowacki, A. S., Jackson, B., & Modlin, C. S. (2018). Health Literacy (HL) & Health-Related Quality of Life (HRQL) Among Minority Men. *Journal of the National Medical Association, 110*(2), 124-129. doi:10.1016/j.jnma.2017.10.001

Myaskovsky, L., Burkitt, K. H., Lichy, A. M., Ljungberg, I. H., Fyffe, D. C., Ozawa, H., . . . Boninger, M. L. (2011). The association of race, cultural factors, and health-related quality of life in persons with spinal cord injury. *Archives of Physical Medicine & Rehabilitation, 92*(3), 441-448. doi:https://dx.doi.org/10.1016/j.apmr.2010.10.007

Omachi, T. A., Sarkar, U., Yelin, E. H., Blanc, P. D., & Katz, P. P. (2011). Lower health literacy among COPD patients is associated with poorer COPD functional outcomes and greater risk of COPD-related emergency utilization. *American Journal of Respiratory and Critical Care Medicine. Conference: American Thoracic Society International Conference, ATS, 183*(1 MeetingAbstracts). Retrieved from http://ajrccm.atsjournals.org/cgi/reprint/183/1_MeetingAbstracts/A2257?sid=9116dfaa-e902-4661-98c2-9c7166e44bd8

Ownby, R. L., Acevedo, A., Waldrop-Valverde, D., Caballero, J., Simonson, M., Davenport, R., . . . Jacobs, R. J. (2017). A Mobile App for Chronic Disease Self-Management: Protocol for a Randomized Controlled Trial. *JMIR research protocols, 6*(4), e53. doi:https://dx.doi.org/10.2196/resprot.7272

Park, N. H., Song, M. S., Shin, S. Y., Jeong, J.-h., Lee, H. Y., & Bardage, B.-L. B (2018). The effects of medication adherence and health literacy on health-related quality of life in older people with hypertension. *International Journal of Older People Nursing, 13*(3), 1-10. doi:https://dx.doi.org/10.1111/opn.12196

Prihanto, J. B., Wahjuni, E. S., Nurhayati, F., Matsuyama, R., Tsunematsu, M., & Kakehashi, M. (2021). Health Literacy, Health Behaviors, and Body Mass Index Impacts on Quality of Life: Cross-Sectional Study of University Students in Surabaya, Indonesia. *International Journal of Environmental Research & Public Health [Electronic Resource], 18*(24), 13. doi:https://dx.doi.org/10.3390/ijerph182413132

Rak, E. C. (2012). Quality of life of persons with diabetes: Understanding the effects of health literacy, self-efficacy and knowledge of chronic illness and disability. *Dissertation Abstracts International: Section B: The Sciences and Engineering, 73*(4-B), 2535. Retrieved from http://ovidsp.ovid.com/ovidweb.cgi?T=JS&PAGE=reference&D=psyc9&NEWS=N&AN=2012-99200-098

Reid, A. L., Porter, K. J., You, W., Kirkpatrick, B. M., Yuhas, M., Vaught, S. S., & Zoellner, J. M. (2021). Low Health Literacy Is Associated With Energy-Balance-Related Behaviors, Quality of Life, and BMI Among Rural Appalachian Middle School Students: A Cross-Sectional Study. *The Journal of school health, 91(8)*, 608-616. doi:http://dx.doi.org/10.1111/josh.13051

Rijken, M., & van der Heide, I. (2019). Identifying subgroups of persons with multimorbidity based on their needs for care and support. *BMC family practice, 20*(1), 179. doi:https://dx.doi.org/10.1186/s12875-019-1069-6

Roberto, L. L., Noronha, D. D., Souza, T. O., Miranda, E. J. P., Martins, A. M. E. B. L., Paula, A. M. B., . . . Haikal, D. S. (2018). Lack of access to information on oral health problems among adults: an approach based on the theoretical model for literacy in health. [Portuguese]. *Ciencia & Saude Coletiva, 23(3)*, 823-835. doi:http://dx.doi.org/10.1590/1413-81232018233.25472015

Rozier, R. G. (2012). Oral health in North Carolina: innovations, opportunities, and challenges. *North Carolina Medical Journal, 73*(2), 100-107. Retrieved from https://ovidsp.ovid.com/ovidweb.cgi?T=JS&CSC=Y&NEWS=N&PAGE=fulltext&D=med9&AN=22860318

Schaffler, J., Leung, K., Tremblay, S., Merdsoy, L., Lambert, S. D., Belzile, E., & Lambrou, A. (2018). The Effectiveness of Self-Management Interventions for Individuals with Low Health Literacy and/or Low Income: A Descriptive Systematic Review. *JGIM: Journal of General Internal Medicine, 33*(4), 510-523. doi:10.1007/s11606-017-4265-x

Scheuer, S. (2018). The patient navigation project for socially vulnerable cancer patients improves quality of life and health literacy. *Journal of Global Oncology, 4(Supplement 2)*, 110s. doi:http://dx.doi.org/10.1200/jgo.18.21300

Simon, M. A., Li, Y., & Dong, X. (2014). Levels of health literacy in a community-dwelling population of Chinese older adults. *Journals of Gerontology Series A-Biological Sciences & Medical Sciences, 69 Suppl 2*, S54-60. doi:https://dx.doi.org/10.1093/gerona/glu179

Stormacq, C., Wosinski, J., Boillat, E., & Den Broucke, S. V. (2020). Effects of health literacy interventions on health-related outcomes in socioeconomically disadvantaged adults living in the community: A systematic review. *JBI Evidence Synthesis, 18(7)*, 1389-1469. doi:https://dx.doi.org/10.11124/JBISRIR-D-18-00023

Talmage, C. A., Figueroa, H. L., & Wolfersteig, W. L. (2018). CULTIVATING A CULTURE OF HEALTH IN THE SOUTHWEST: LINKING ACCESS AND SOCIAL DETERMINANTS TO QUALITY OF LIFE AMONGST DIVERSE COMMUNITIES. *Journal of Health and Human Services Administration, 40*(4), 397-432. Retrieved from https://www.scopus.com/inward/record.uri?eid=2-s2.0-85049038194&partnerID=40&md5=9b94d71623c53dda0b9d50ff3f05158e

Tan, S. S., Pisano, M. M., Boone, A. L. D., Baker, G., Pers, Y. M., Pilotto, A., . . . Effichronic, C. (2019). Evaluation Design of EFFICHRONIC: The Chronic Disease Self-Management Programme (CDSMP) Intervention for Citizens with a Low Socioeconomic Position. *International journal of environmental research and public health, 16*(11). doi:10.3390/ijerph16111883

Todorovic, N., Jovic-Vranes, A., Djikanovic, B., Pilipovic-Broceta, N., Vasiljevic, N., Lucic-Samardzija, V., & Peric, A. (2019). Assessment of health literacy in the adult population registered to family medicine physicians in the Republic of Srpska, Bosnia and Herzegovina. *European Journal of General Practice, 25*(1), 32-38. doi:10.1080/13814788.2019.1571579

Uddin, M. N., Bhar, S., Al Mahmud, A., & Islam, F. M. A. (2017). Psychological distress and quality of life: rationale and protocol of a prospective cohort study in a rural district in Bangaladesh. *BMJ Open, 7*(9), e016745. doi:https://dx.doi.org/10.1136/bmjopen-2017-016745

Virlée, J., van Riel, A. C. R., & Hammedi, W. (2020). Health literacy and its effects on well-being: how vulnerable healthcare service users integrate online resources. *Journal of Services Marketing, 34*(5), 697-715. doi:10.1108/JSM-02-2019-0057

Walker, R. J., Gebregziabher, M., Martin-Harris, B., & Egede, L. E. (2014). Independent effects of socioeconomic and psychological social determinants of health on self-care and outcomes in Type 2 diabetes. *General Hospital Psychiatry, 36*(6), 662-668. doi:https://dx.doi.org/10.1016/j.genhosppsych.2014.06.011

Wang, C., Kane, R. L., Xu, D., & Meng, Q. (2015). Health literacy as a moderator of health-related quality of life responses to chronic disease among Chinese rural women. *BMC Women's Health, 15*, 34. doi:https://dx.doi.org/10.1186/s12905-015-0190-5

Wang, C., Li, H., Li, L., Xu, D., Kane, R. L., & Meng, Q. (2013). Health literacy and ethnic disparities in health-related quality of life among rural women: results from a Chinese poor minority area. *Health & Quality of Life Outcomes, 11*, 153. doi:https://dx.doi.org/10.1186/1477-7525-11-153

Washington, D. M., Curtis, L. M., Waite, K., Wolf, M. S., & Paasche-Orlow, M. K. (2018). Sociodemographic Factors Mediate Race and Ethnicity-associated Childhood Asthma Health Disparities: a Longitudinal Analysis. *Journal of Racial & Ethnic Health Disparities, 5*(5), 928-938. doi:https://dx.doi.org/10.1007/s40615-017-0441-2

Webb, J. (2020). Social aspects of chronic transfusions: addressing social determinants of health, health literacy, and quality of life. *Hematology, 2020*(1), 175-183. doi:https://dx.doi.org/10.1182/hematology.2020000104

White, B. M., Hall, E. S., & Johnson, C. (2014). Environmental health literacy in support of social action: an environmental justice perspective. *Journal of Environmental Health, 77*(1), 24-29. Retrieved from https://ovidsp.ovid.com/ovidweb.cgi?T=JS&CSC=Y&NEWS=N&PAGE=fulltext&D=med11&AN=25185324

Wong Min, F., Hazreen Abdul, M., Rozmi, I., Tin Tin, S., Tan Maw, P., & Mas Ayu, S. (2022). Psychosocial factors associated with mental health and quality of life during the COVID-19 pandemic among low-income urban dwellers in Peninsular Malaysia. *medRxiv*. doi:10.1101/2022.02.21.22271310

Xiao, Z., Lee, J., & Liu, W. (2020). Korean and Vietnamese immigrants are not the same: Health literacy, health status, and quality of life. *Journal of Human Behavior in the Social Environment, 30*(6), 711-729. doi:10.1080/10911359.2020.1740852

Xu, R. H., Zhou, L. M., Wong, E. L. Y., & Wang, D. (2021). The association between patients' ehealth literacy and satisfaction with shared decision-making and well-being: Multicenter cross-sectional study. *Journal of medical Internet research, 23(9) (no pagination)*. doi:http://dx.doi.org/10.2196/26721

Aaby, A., Friis, K., Christensen, B., & Maindal, H. T. (2020). Health Literacy among People in Cardiac Rehabilitation: Associations with Participation and Health-Related Quality of Life in the Heart Skills Study in Denmark. *International Journal of Environmental Research & Public Health [Electronic Resource], 17*(2), 09. doi:https://dx.doi.org/10.3390/ijerph17020443
